# Transient Receptor Potential Vanilloid 6 (TRPV6) Proteins Control the Extracellular Matrix Structure of the Placental Labyrinth

**DOI:** 10.3390/ijms21249674

**Published:** 2020-12-18

**Authors:** Manuel Winter, Petra Weissgerber, Karolin Klein, Femke Lux, Daniela Yildiz, Ulrich Wissenbach, Stephan E. Philipp, Markus R. Meyer, Veit Flockerzi, Claudia Fecher-Trost

**Affiliations:** 1 Experimental and Clinical Pharmacology and Toxicology, Center for Molecular Signaling (PZMS), Saarland University, Buildings 61.4 and 46, 66421 Homburg, Germany; manuel.winter@uks.eu (M.W.); petra.weissgerber@uks.eu (P.W.); KaroKlein@msn.com (K.K.); Femke.Lux@uks.eu (F.L.); Daniela.Yildiz@uks.eu (D.Y.); Ulrich.wissenbach@uks.eu (U.W.); Stephan.Philipp@uks.eu (S.E.P.); m.r.meyer@mx.uni-saarland.de (M.R.M.); veit.flockerzi@uks.eu (V.F.); 2 Transgenic Technologies, Center for Molecular Signaling (PZMS), Saarland University, Building 61.4, 66421 Homburg, Germany; 3 Zentrum für Human- und Molekularbiologie (ZHMB) and Center for Molecular Signaling (PZMS), Saarland University, Buildings 61.4 and 46, 66421 Homburg, Germany

**Keywords:** TRPV6, calcium homeostasis, trophoblast, extracellular matrix, placenta, fibronectin, FND3A, HTRA1, granzyme, nano LC–MS/MS

## Abstract

Calcium-selective transient receptor potential Vanilloid 6 (TRPV6) channels are expressed in fetal labyrinth trophoblasts as part of the feto–maternal barrier, necessary for sufficient calcium supply, embryo growth, and bone development during pregnancy. Recently, we have shown a less- compact labyrinth morphology of *Trpv6-*deficient placentae, and reduced Ca^2+^ uptake of primary trophoblasts upon functional deletion of TRPV6. *Trpv6^-/-^* trophoblasts show a distinct calcium-dependent phenotype. Deep proteomic profiling of *wt* and *Trpv6^-/-^* primary trophoblasts using label-free quantitative mass spectrometry leads to the identification of 2778 proteins. Among those, a group of proteases, including high-temperature requirement A serine peptidase 1 (HTRA1) and different granzymes are more abundantly expressed in *Trpv6^-/-^* trophoblast lysates, whereas the extracellular matrix protein fibronectin and the fibronectin-domain-containing protein 3A (FND3A) were markedly reduced. *Trpv6^-/-^* placenta lysates contain a higher intrinsic proteolytic activity increasing fibronectin degradation. Our results show that the extracellular matrix formation of the placental labyrinth depends on TRPV6; its deletion in trophoblasts correlates with the increased expression of proteases controlling the extracellular matrix in the labyrinth during pregnancy.

## 1. Introduction

Ca^2+^ transport through the transient receptor potential vanilloid 6 (TRPV6) channel across the feto–maternal barrier plays a pivotal role in embryonic development and bone development [1]. Calcium ions act as secondary messengers and are key players in many steps of reproduction, starting with e.g., sperm maturation [2,3], uterus conversion processes, placenta development, and in-term labor. The cytosolic calcium concentration (~100 nM) is tightly regulated by calcium transporters, calcium permeable ion channels, and proteins acting as calcium buffers and Ca^2+^-dependent signaling molecules [4]. Placental transport of calcium to the fetus increases massively towards the third trimester of pregnancy to fulfill the increasing requirement of the embryonic bone development. Accompanying this, *Trpv6* gene expression in mice is strongly increases from embryonic (E) day E15 to E18 [5,6].

During placental development, trophoblast cells form the interface between maternal and fetal circulation. Trophoblasts are involved in ion homeostasis and many specialized functions like migration, vascular remodeling, and hormone secretion [7,8,9]. Changes in the cytosolic (Ca^2+^) of trophoblasts are involved in the regulation of these processes, and knowledge of the role of calcium-conducting TRP channels is steadily increasing [10]. For example, the Ca^2+^-selective TRPV6 channel is expressed during pregnancy [11,12,13] in fetal and maternal structures; in the trophoblasts of the fetal labyrinth, the maternal decidua, and the yolk sac [1]. Embryo growth and bone mineralization is dependent on TRPV6 and is significantly reduced in placentae deficient in *Trpv6* as a result of reduced calcium uptake [1]. This effect continues through day E14.5 and was much more pronounced in embryos in which both *Trpv6* alleles of the maternal placenta were simultaneously deleted. Male *Trpv6^-/-^* or *Trpv6^mt/mt^* animals are hypofertile and therefore heterozygous males must be mated with heterozygous or homozygous *Trpv6^-/-^* females [2,3]. The genotype of the maternal part of the placenta is responsible for the pronounced effect on the bone mineralization because the offspring of homozygous *Trpv6-*deficient females are much more affected than the offspring from heterozygous females [1]. This is consistent with case reports in humans, where mutations in the *Trpv6* gene lead to the hereditary human disease transient neonatal hyperparathyroidism (HRPTTN, OMIM #618188) associated with skeletal abnormalities, dysplasia, and elevated neonatal parathyroid hormone levels [14,15,16,17,18]. Those authors conclude, that similar to the *Trpv6* mouse model [1], *Trpv6* mutations in the maternal and fetal parts of the placenta greatly reduce maternal/fetal calcium transport, thereby affecting infant skeletal development and mineralization. Homozygous murine *Trpv6^-/-^* embryos developing into *Trpv6^-/-^* mothers accumulated less calcium, had reduced bone mineralization and altered bone biomechanics that persisted into adulthood [1]. Key initial observations in the placenta of pregnant mice were that both the deletion of the *Trpv6* gene (*Trpv6^-/-^*) and the functional inactivation of the TRPV6-ion-conducting pore by a point mutation (*Trpv6^mt/mt^*) led to morphological changes of the placental labyrinth, a reduction of Ca^2+^ accumulation in the embryo up to E14.5, and a reduced Ca^2+^ uptake by *Trpv6-*deficient trophoblasts isolated from these animals. 

To characterize the possible relationship between trophoblast function and morphology of the placental labyrinth, we have now examined trophoblasts, acutely isolated from *Trpv6^-/-^*, *Trpv6^mt/mt^,* and wild-type placentae, for viability, growth, and migration behavior and have characterized their proteome by mass spectrometry. We can show that the amounts of proteases in *Trpv6-*deficient trophoblasts are significantly increased while the amounts of fibronectin and fibronectin type-III domain-containing protein 3A (FND3A) are decreased. In particular, fibronectin, as a structural glycoprotein in the extracellular tissue matrix, mediates cell–cell, cell–matrix, and cell–substrate adhesion and an increased intrinsic proteolytic activity of trophoblasts could interfere with these functions and be responsible for the altered morphology of the placental labyrinth. 

## 2. Results

### 2.1. Calcium-Dependent Cell Growth, Viability, and Migration Behavior of wt and Trpv6^-/-^ Trophoblast Cells

**Figure 1:**
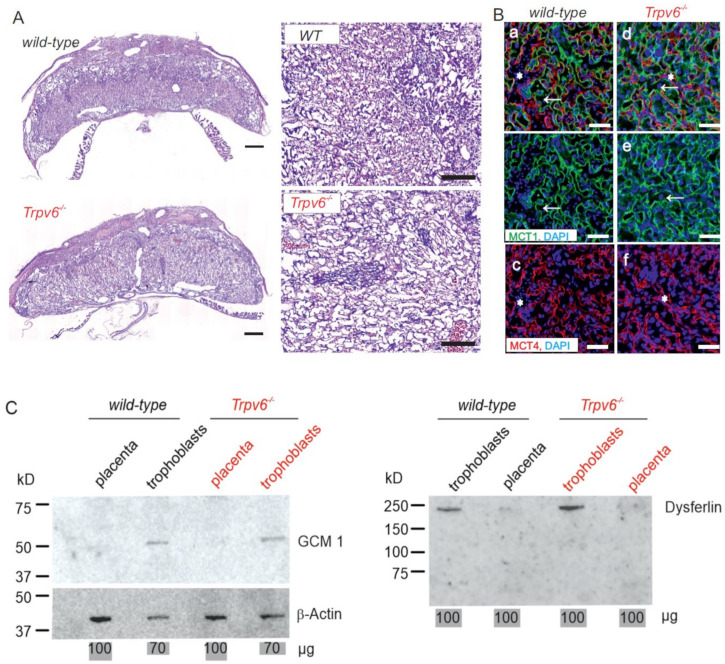
The *Trpv6^-/-^* placental labyrinth structure is less compact. (**A**) Hematoxylin–eosin staining of murine wild-type and *Trpv6^-/-^* placenta sections (left) and two enlarged sections from two other placentae of the labyrinth zone (right) at E14.5 are shown. Scale bar: placenta (left), 500 µm; labyrinth zone (right), 200 µm. (**B**) Immunostaining of the placental labyrinth zone from *wt* and *Trpv6^-/-^* at E14.5 with antibodies against monocarboxylate transporters 1 and 4 (MCT1, MCT4), the marker proteins of syncytiotrophoblast layers 1 and 2, respectively. The immunoreactivity for MCT1 (green signal) was detected in syncytiotrophoblast I cell layer which faced the maternal blood sinuses (arrow) and for MCT4 (red signal) in syncytiotrophoblast II cells which faced the fetal blood vessels (*), blue, DAPI stain, scale bar = 50 µm. (**C**) Western blot of protein lysates from 100 µg placenta and 70 µg trophoblast were tested for the presence of the trophoblast marker chorion-specific transcription factor GCMa (GCM1) and dysferlin. Antibody against beta-actin was used as loading control.

We confirmed our previous results [1] that the morphology of the wt and Trpv6^-/-^ labyrinth zone in the mouse placenta was different; the Trpv6^-/-^ labyrinth was less dense and has much larger cell-free spaces (Figure 1A), which in turn could indicate that the cell structures where maternal–fetal exchange appears are somehow affected. In order to characterize the labyrinth morphology, we first asked whether there is different distribution of the two labyrinth syncytiotrophoblasts layers I+ and II (SynTI + SynTII). Immunostaining of representative labyrinth regions of wt and Trpv6^-/-^ placentae was done using antibodies directed against the monocarboxylate transporters MCT1 and MCT4. MCT1 is specifically expressed at the apical side of SynTI and MCT4 at basal side of SynTII membranes facing to the maternal and fetal blood circulation, respectively [19,20]. We detected no obvious structural difference in the SynTI and SynTII layer distribution between the wt and Trpv6^-/-^ genotypes at E14.5 (Figure 1B). 

**Figure 2:**
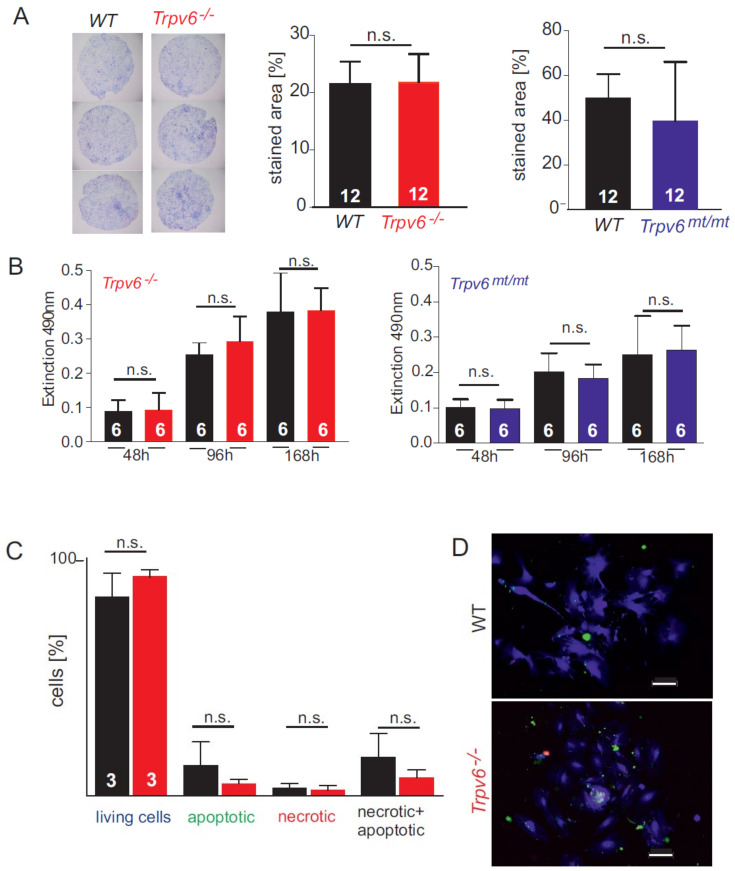
Cell viability and migration behavior of trophoblasts do not depend on the *Trpv6* genotype. (**A**–**D**), *wt*, *Trpv6^-/^,^-^* and *Trpv6^mt/mt^* were isolated from placentae. (**A**) left side: bright field pictures from the bottom side of transwell membranes (8 µm pores) stained with eosin/azur to visualize migrated trophoblast cells after 48-h incubation. Middle and right side: data are presented as % of stained area + SD (*n* = 12 for each genotype, respectively) for *Trpv6^-/-^* (red) and *Trpv6^mt/mt^* (blue) as compared to *wt* (black). (**B**) Quantitative measurement of cell viability over 7 days shown as the formazan extinction (at 490 nm). The cell activity of three independent preparations was measured in wild-type trophoblasts (black) and *Trpv6^-/-^* (red) and *Trpv6^mt/mt^* (blue) trophoblast cells on days 2, 4, and 7 after isolation (number of dishes, *n* = 6). (**C**) Recording of % living (blue), apoptotic (green), and necrotic (red) cells in comparison of wild-type and *Trpv6^-/-^* trophoblasts (*n* = 3 independent cell isolations, 28 and 18 microscopic pictures from *wt* and *Trpv6^-/-^* trophoblasts were analyzed, respectively). (**D**) Microscopic picture of living (blue), apoptotic (green), and necrotic (red) cells stained after 72 h in culture, scale bar = 100 µm. (**A**–**C**) The significance of the differences between groups was calculated using Student’s *t*-test (not significant ((n.s.).

Next, we isolated primary wt, Trpv6^mt/mt^ and Trpv6^-/-^ trophoblasts by percoll gradient centrifugation (Supplementary Figure S1). The trophoblast marker proteins chorion-specific transcription factor GCMa (GCM1) and dysferlin, were markedly enriched in the trophoblast cell fraction from both genotypes and less detectable in whole-placenta lysates (Figure 1C). Trophoblasts were seeded on a transwell chamber and the migrated cells at the bottom side of the membrane were stained after 48 h incubation with eosin/azur (Supplementary Figures S1 and S2A). Cell migration of trophoblasts of the three different genotypes was not different. The viability of trophoblast cells in culture medium supplemented with 1.5 mM calcium was also not different between wt, Trpv6^mt/mt^, and Trpv6^-/-^ (Figure 2B). Likewise, the number of living, necrotic, and apoptotic cells was also not changed between wt and Trpv6^-/-^ concluding that the absence or presence of functional TRPV6 protein has no influence on the viability of trophoblast cells cultured with 1.5 mM calcium (Figure 2C,D). 

**Figure 3:**
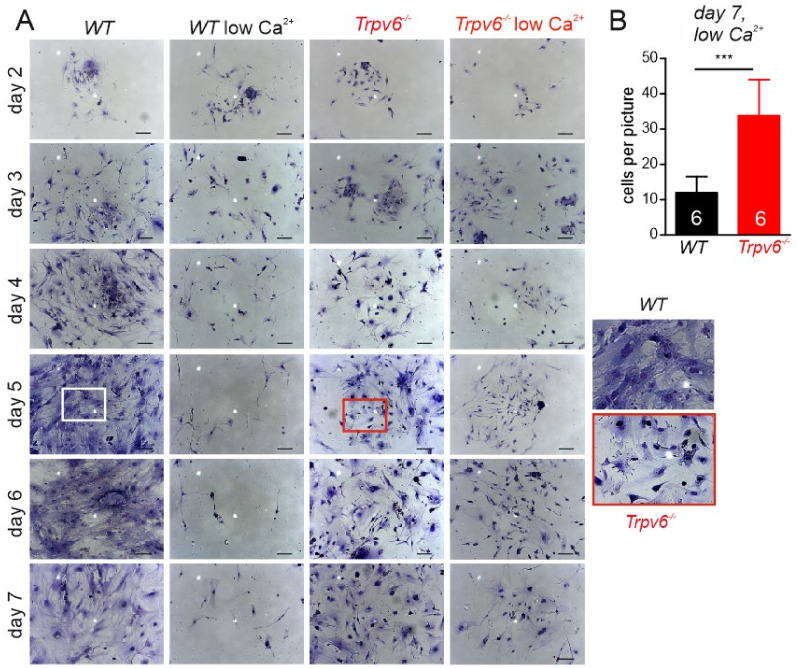
Primary cell culture of *wt* and *Trpv6^-/-^* trophoblasts (E14.5). (**A**) *Wild-type* and *Trpv6^-/-^* trophoblasts (200,000 per dish) were cultured with medium in the presence of 1.5 mM calcium. At day 2 of cell culture the calcium concentration was reduced to 0.1 mM free calcium concentration (*wt* low Ca^2+^, *Trpv6^-/-^* low Ca^2+^). Cells in normal or reduced calcium were cultured over seven days. Cells were fixed and stained with eosin/azur stained. Scale bar: 100 µm. Insets (red: *Trpv6^-/-^,* white: *wt*) of stained trophoblast cells in culture at day 5. (**B**) After 7 days, cells cultured in low calcium medium were fixed, stained with eosin/azur and adherent cells were counted (*n* = 6 pictures from *N* = 2 independent preparations). The significance of the differences between wt and *Trpv6^-/-^* was calculated using Student’s *t*-test (*** *p* < 0.001), Results are shown as mean + SD.

In further experiments we cultured primary trophoblasts in medium with normal (1.5 mM) or low (0.1 mM) calcium concentrations for seven days (Figure 3). On day two wt trophoblasts started to spread extensively and develop a dense extracellular network until day 5. Upon calcium reduction to 0.1 mM in the culture medium at day 2, wt cells rapidly began to die keeping only a small number alive after seven days. In contrast to *wt*, *Trpv6*-deficient trophoblasts built fewer and shorter protrusions in the presence of high calcium, and did not develop an equally-dense extracellular matrix (ECM). These morphological differences suggested that, the extracellular matrix assembly depended on the TRPV6 channel function. In addition, a fraction of Trpv6^-/-^ cells died in the low calcium medium but more Trpv6^-/-^ trophoblasts survived after seven days (Figure 3B), indicating that these cells contained mechanisms to compensate low calcium conditions.

### 2.2. Proteome Analysis of Primary Trophoblasts from wt and Trpv6^-/-^ Placentae at E14.5

In order to identify the individual proteomic profiles of wt and Trpv6^-/-^ trophoblasts with a label-free mass spectrometric approach, cells were isolated from wt and Trpv6^-/-^ placentae by filtration and percoll gradient centrifugation (Supplementary Figure S1), and equal protein amounts were separated on SDS-PAGE gels. In total, eight wt and eight Trpv6^-/-^ trophoblast cell lysates were analyzed by nano LC–MS/MS (Supplementary Figure S2 and S3). By this approach 2778 proteins in all lysates for both genotypes were identified. The overall number of identified proteins for wt and Trpv6^-/-^ trophoblasts was 2597 and 2181, respectively. Semiquantitative analysis of data from both genotypes demonstrated that in Trpv6^-/-^ trophoblasts 35 proteins were significantly more abundant while 314 proteins were downregulated compared to the wt control (Supplementary Figures S2 and S3A,B). 

### 2.3. Proteases Are More-Abundantly Expressed in TRPV6-Deficient Trophoblasts 

**Figure 4:**
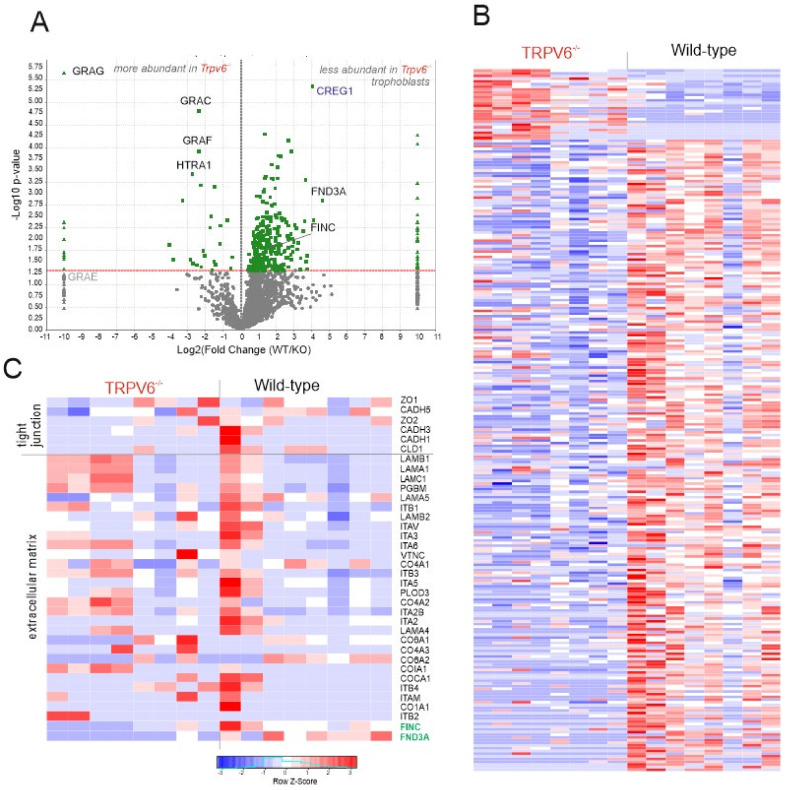
Proteome analysis of *wt* and *Trpv6^-/-^* trophoblasts. (**A**) Vulcano-blot-reporting semi-quantitative analysis of differentially-expressed proteins identified by mass spectrometry in *wild-type* and *Trpv6^-/-^* trophoblast lysates. Shown are 35 proteins upregulated and 314 proteins down-regulated in *Trpv6^-/-^* compared to *wild-type* controls. Up- and down-regulated proteins were identified based on at least 2-fold changes in the exponentially-modified protein abundance index (emPAI) values with a *p*-value < 0.05, calculated using the unpaired two-tailed Student’s *t*-test. Proteins identified on the very left or the very right side of the blot (−10,10) were exclusively detected in *Trpv6^-/-^* and *wt* trophoblasts, respectively. Fibronectin-domain-containing protein 3A (FND3A) and fibronectin (FINC) (green) and CREG1 (blue) were significantly more abundant in *wt* lysates. (**B**) Heatmap shows the Z-scores of the emPAI values from independent mass spectrometry measurements from eight independent wild-type and *Trpv6^-/-^* samples. (**C**) Heatmap shows Z-scores for emPAI values of identified proteins detected in the gene ontology terms tight junctions and extracellular matrix/assembly. Fibronectin-domain-containing protein 3A (FND3A) and fibronectin (FINC) (green) were significantly more abundant in *wt* lysates.

**Figure 5:**
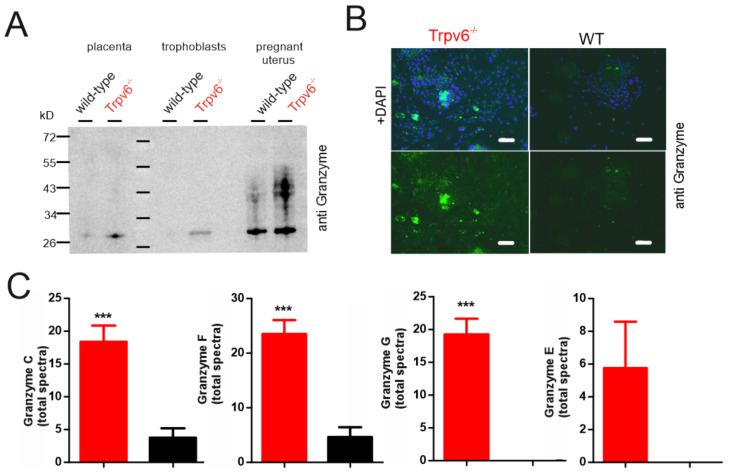
Granzymes are more abundantly expressed in *Trpv6^-/-^* trophoblasts. (**A**) Western blot of protein lysates obtained at day of preparation (day1) from placentae, trophoblasts and pregnant uteri were tested for the presence of granzymes. Equal amounts of proteins, as determined by bicinchoninic acid (BCA) assay were loaded. (**B**) Detection of granzymes in trophoblast cells at day 3 of cell culture, bar = 100 µm. (**C**) MS/MS-identified spectra of different granzyme isoforms identified in *wild-type* and *Trpv6*-deficient trophoblasts (*n* = 8 lysates/genotype). The significance difference between the groups was calculated using Student’s *t*-test (*** *p* < 0.001).

The amount of proteases, including four granzymes (G, C, E, and F) and the high-temperature requirement A serine peptidase 1 (HTRA1) were exclusively- or significantly-more abundant in the Trpv6^-/-^ trophoblast cells (Figure 4A,B and Supplementary Table S1). HTRA1 is highly expressed in placentae and contains trypsin-like protease domains [21,22]. Granzymes are a family of endopeptidases with intra- and extracellular targets, mainly in the immune system, regulating apoptosis and inflammation [23]. With the proteomic approach, we did not detect any of the prominent Granzymes A or B (Supplementary Table S1) in the trophoblast lysates. Similarly Ncr1 and Klrb1c, marker proteins of NK cells and CD3 and CD19, marker proteins of B- and T-cells were also not detectable [24,25]. Likewise the purity check of the enriched trophoblast fraction from wt placentae by Fluorescence Activated Cell Sorting (FACS) analysis, indicted a negligible impurity by NK cells (0.036% of the total cell fraction, Supplementary Figure S4). In sum the data indicated that NK cells were probably not included in the trophoblast preparation and were therefore not the origin of granzyme expression. The amount of granzymes present in the placenta, pregnant uterus, and trophoblasts were compared on the day of isolation by Western blot using a pan-granzyme antibody (Figure 5A). The pan-granzyme antibody was suitable to detect different granzyme isoforms, like B, C, E , or F, which are very homolog in the central region. According to the mass spectrometrical results we detected a higher amount of “granzymes” in Trpv6-deficient trophoblasts (Figure 5A,B,C and Supplementary Table S1). The more intense Western blot signals in the placenta and especially the pregnant uterus at E14.5 were presumably caused by granzymes abundantly expressed in NK cells of the pregnant uterus [26]. With the mass spectrometry different granzyme isoforms were detected in Trpv6-deficient trophoblasts (Figure 5C and Supplementary Table S1); granzyme G spectra were exclusively detected in Trpv6^-/-^ trophoblasts, granzymes C and F were significantly enriched and the peptide spectra for granzyme E were identified in three of eight Trpv6^-/-^ trophoblast preparations (and therefore not significantly enriched). Accordingly, a higher granzyme expression level could be detected in the Trpv6^-/-^ trophoblast cell culture three days after isolation (Figure 5B), demonstrating that the granzyme C, E, F, and G proteases are more abundant in the TRPV6-deficient labyrinth trophoblasts. 

### 2.4. The amount of Extracellular Matrix Proteins Is Reduced in TRPV6-Deficient Trophoblasts

**Figure 6:**
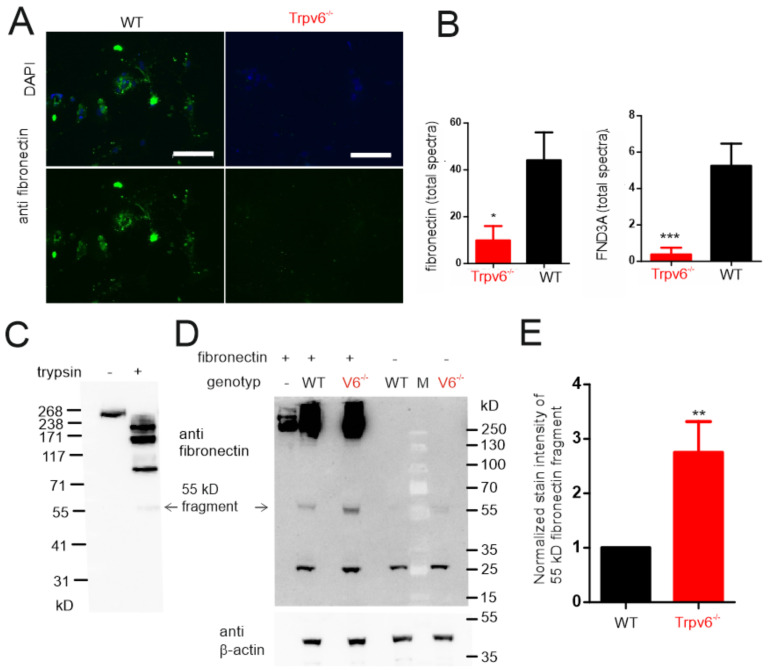
Fibronectin and FND3A are more abundantly expressed in wt trophoblasts (**A**) Detection of fibronectin in trophoblast cells at day 3 of cell culture after isolation from placentae with anti-fibronectin antibody, bar = 100 µm. (**B**) MS/MS-identified spectra of fibronectin and FND3A protein identified in wild-type and transient receptor potential vanilloid 6 (Trpv6)-deficient trophoblasts (*n* = 8 lysates/genotype). The significance difference between groups was calculated using Student’s *t*-test (* *p* < 0.05, ** *p* < 0.01, *** *p* < 0.001). (**C**) Western blot of tryptic-digested and non-digested fibronectin with a fibronectin antibody. (**D**) Western blot analysis of a protease assay with placenta lysates. Placenta lysates from wt and Trpv6^-/-^ were incubated with fibronectin for 1 h and fibronectin fragments were detected with anti-fibronectin antibody. A beta-actin antibody was used as loading control. (**E**) Bar graph shows the normalized intensity of the proteolytic fibronectin fragment detected in wt and Trpv6^-/-^ placenta lysates by Western blot analysis in *n* = 3 experiments. Results are shown as mean + SD.

Granzymes cleave both extracellular and intracellular proteins [27]. Because the morphology of Trpv6^-/-^ trophoblast cell cultures is less dense and smaller compared to the wt cells (Figure 3), we analyzed the protein levels of typical extracellular matrix proteins, like integrins, basement membrane proteoglycan, vitronectin, laminins, vinculin or collagens (Figure 4C). The amounts of these typical extracellular matrix proteins were not significantly different between genotypes (Supplementary Table S1 and Figure 4C) except for fibronectin. Four times fewer peptide spectra were identified for fibronectin in the Trpv6^-/-^ cells at the day of trophoblast isolation (Figure 4A,C) and also a reduced protein amount on day 3 in cell culture in comparison to wt cells (Figure 6A,B). Fibronectin is an adaptor connecting integrins with the cellular matrix. It is a highly-glycosylated adhesion protein that binds from the extracellular side to integral integrin receptors and connects various extracellular secreted proteins to the cell surface. We also detected domain-containing protein 3A (FND3A, gene: FNDC3A), also involved in cell adhesion and ECM assembly [28]. Thirteen-times-more FND3A peptides were identified in the wild-type trophoblasts (Figure 4C and Figure 6B, and Supplementary Table S1). 

In contrast to the proteins fibronectin and FND3A, which are involved in the extracellular matrix and are necessary for formation and assembly, we did not detect changes for the protein amounts belonging to the group of tight junction proteins, such as claudins, cadherins, or zona occludens proteins 1 and 2 (Figure 4C and Supplementary Figure S3). One protein that appeared to control cell adherence is the protein cellular repressor of E1A-stimulated gene (CREG1), which was 16-times-less abundant in Trpv6^-/-^ trophoblast cells than in trophoblast cells isolated from wild-type placentae (Figure 4A, blue, and Supplementary Table S1). CREG1 is necessary for the differentiation of mouse embryonic stem cells into cardiomyocytes, is enriched at cell–cell junctions in cardiomyocyte cell cultures and enhances the assembly of gap junctions when overexpressed [29].

Additional to the granzyme C, E, F, and G proteases, the high-temperature requirement A1 serine protease (HTRA1) was significantly more abundant in the Trpv6^-/-^ trophoblasts (Figure 4C and Supplementary Table S1). HTRA1 digests also extracellular matrix proteins, like fibronectin, decorin, biglycan, fibromodulin, and collagens [30,31]. In summary our experiments demonstrated that isolated Trpv6-deficient trophoblast preparations contain larger amounts of serine proteases and contain a lower amount of fibronectin and FND3A. 

### 2.5. Increased Proteolytic Activity in Trpv6^-/-^ Placentae Causes Increased Fibronectin Degradation

During the proteome analysis we identified fewer fibronectin spectra in Trpv6^-/-^ trophoblast lysates. Likewise, minor fibronectin could be detected with fibronectin antibody staining in Trpv6^-/-^ trophoblast cells when they were cultured for three days (Figure 6A). Finally, we tested, in protease activity assays, whether there is a causal link between increased amounts of proteases and decreased fibronectin in Trpv6^-/-^ trophoblasts. As a proof of principle, we first incubated fibronectin in the presence of the serine protease trypsin and visualized fibronectin fragments by Western blot. As shown in Figure 6C, fibronectin (MW 273 kD) was cleaved by trypsin under the given conditions into smaller fragments. Next, we incubated fibronectin with lysates from wild-type or Trpv6-deficient placentae and visualized fibronectin fragments by Western blot (Figure 6D,E). A smaller 55 kD fibronectin fragment was also detectable in higher amounts when fibronectin was incubated with the Trpv6-deficient lysates. This suggests that the Trpv6-deficient placentae have a higher intrinsic proteolytic activity. At the same time these results correlate with the mass spectrometry results, where more proteases and less fibronectin were detected in Trpv6^-/-^ trophoblasts (Figure 4, Figure 5, and Figure 6A,B).

### 2.6. What Are the Potential Mechanisms Underlying Higher Protease Expression and Activity in Trpv6- Deficient Placentae? 

As we have shown [1], *Trpv6-*deficient trophoblasts contain less calcium in comparison to *wt* trophoblasts. Calcium ions stabilize proteins against thermal and proteolytic degradation, thereby inhibiting, for example, the autolysis of trypsin-like serine proteases [32]. It is possible that the lower calcium content of *Trpv6^-/-^* trophoblasts makes it easier to activate the proteases in these cells. Another possibility is that the expression of the proteases is controlled by calcium-dependent transcription factors [33]. The protein amounts of the calcium-dependent protease calpain1 and 2 (CAN1, CAN2) are not different in WT and *Trpv6^-/-^* trophoblasts, whereas the amount of the small regulatory calpain subunit (CPNS1) is higher in WT trophoblasts than in *Trpv6^-/-^* trophoblasts, that contain less intracellular calcium (Supplementary Table S1). It is also conceivable that TRPV6 is involved in crosstalk between trophoblasts and NK cells that control the secretion of granzymes and cytokines [24,34]. Co-cultures of NK-immune cells and trophoblasts could help to investigate this possibility. 

Taken together, we can show that a functional deletion of TRPV6 in trophoblasts correlates with the increased expression of proteases that limit the ECM formation in the labyrinth during pregnancy.

## 3. Discussion

Trpv6-deficient trophoblasts expressed higher amounts of protease (HTRA1 and granzyme C, E, F, and G) which promote degradation of fibronectin in the extracellular matrix.

The proteomic profiling of *wt* and *Trpv6^-/-^* placental trophoblasts led to the identification of 2778 proteins, with 35 proteins being significantly-more abundant in *Trpv6*-deficient trophoblasts. Among those the proteases granzyme C, E, F, and G and HTRA1 were markedly upregulated. Granzymes are a family of structurally-related serine proteases that differ in their substrate specificity. Five human granzymes have been described—A, B, H, K, and M [35] and eleven isoforms in mice, including A and B and mouse-specific granzymes C, D, E, F, and G [36]. The murine granzyme C seems the most probable orthologue of human granzyme H. We did not detect any granzyme A and B by mass spectrometry in the *wt* and *Trpv6^-/-^* trophoblast lysates. Types A and B are the major granzymes expressed in cytotoxic T lymphocyte (CTL) and natural killer (NK) cells [27,37]. NK cells are among the immune cells in the pregnant uterus. The granzymes C, E, F, and G are exclusively expressed in mouse cells and tissues, not in humans and only little information is available on their physiological functions. A delayed expression of granzymes D, E, F, and G has been reported in mouse granulated metrial gland cells, which are uterine NK cells [26]. Whereas granzyme A is expressed in early- to mid-gestation, the expression of granzymes D, E, F, and G is highest in mid- to late gestation and depends on interleukin-2 expression. These results suggest that granzymes D, E, F, and G may have different functions than granzyme A and B during pregnancy. Our data identified negligible amounts of NK cells (0.036%) in the trophoblast cell preparation. We therefore assume that the trophoblast cells express the identified granzymes C, E, F, and G in the third trimester of gestation. Granzyme B has already been detected in the human term syncytiotrophoblast layer and in human Sertoli cells [38]. The expression in the syncytiotrophoblast layer could indicate that granzyme B has an immune-response-independent function and could for example, contribute to the reorganization of the extracellular matrix in the human placenta during pregnancy. Additionally, transcripts of granzymes D, E, F, and G are strongly upregulated in mouse giant trophoblasts after leptin treatment, which indicates that giant trophoblasts could express these granzyme isoforms after certain stimuli [39]. 

Besides the granzymes C, G, E, and F, the serine protease HTRA1 is strongly upregulated in *Trpv6^-/-^* trophoblasts. *Htra1* knock-out mice have a placental phenotype with smaller placentae and intrauterine growth retardation; the pups are too small for their gestational age [40]. In humans high concentrations of the HTRA1 protein in the placenta and serum of preeclampsia patients have been reported, [41,42,43]. These results imply that HTRA1 expression and activity contribute to proper placental function.

Both, the granzymes and HTRA1 cleave proteins of the extracellular matrix, including fibronectin. We found a higher proteolytic activity in *Trpv6^-/-^* placenta lysates with respect to fibronectin cleavage (Figure 6). This correlates with the results of the proteome analysis, where four-times-less fibronectin was detected in *Trpv6^-/-^* trophoblasts (Figure 4 and Figure 6B), and the immune staining of cell cultures, where after three days the protein amount of fibronectin was significantly reduced (Figure 6A). As an adapter protein, fibronectin connects the integrin receptors of the plasma membrane with the extracellular proteins [44,45,46] and is involved in cell adhesion, cell motility, opsonization, wound healing, and cell-shape maintenance [45,46,47]. 

By mass spectrometry we also detected the FND3A protein, which has been reported to influence the formation of the ECM network. Thus, 13-times-more FND3A peptides were identified in the wild-type trophoblasts (Figure 6B) which could be an indicator for the involvement of FND3A proteins in modelling the extracellular matrix of trophoblasts. For example, in *Fndc3a-*deficient zebrafish, changes in the extracellular matrix occur with defects in the fin development and regeneration [48]. The FND3A protein contains nine fibronectin type III domains and is necessary for cell adhesion between spermatids and Sertoli cells, while functional FND3A deletion in mice led to sterility [49]. From our results we conclude that decreased amounts of fibronectin and FND3A reduce the extracellular matrix in *Trpv6^-/-^* trophoblast cell culture (Figure 2) in comparison to *wild-type* cell culture. 

## 4. Materials and Methods 

### 4.1. Mice

Mice were killed and taken for organ removal, according to guidelines of Animal Welfare Law of 18 May 2006 (last change 26 June 2014, § 4 section 3). Guidelines were supervised by the animal welfare officer of the Saarland University. Mice were kept under a standard light/dark cycle with food and water ad libitum. *Trpv6^-/-^* and *Trpv6^mt/mt^* mice are described in [2,3]. 

### 4.2. Isolation of Primary Trophoblast Cells from Pregnant Mice and Cell Culture

Pregnant control “wild-type” mice (matings from female x male, 129 SVJ x C57Bl6N) and pregnant female *Trpv6^-/-^* mated with male *Trpv6^+/-^* were euthanized at embryonic (E) day E14.5 and the uteri were dissected. This mating strategy was necessary because *Trpv6^-/-^* males are hypofertile [2]. Each individual fetus was removed from its amniotic sac, the neck was incised, fetus and fetal membrane were removed and placentae were isolated according to [50]. A small piece of the amniotic sac was removed for genotyping [1]. Next, 7−15 placentae of each genotype were collected in 25 mL ice-cold dissociation buffer (1x Medium199 (Sigma Aldrich, Darmstadt, Germany), 0.02 M Hepes, 0.01 M sodium bicarbonate, 100 µg/mL penicillin−streptomycin (Sigma Aldrich)) containing 20 U/mL DNase I (Roche, Mannheim, Germany), and 125 U/mL collagenase I (Gibco-Invitrogen, Schwerte, Germany) and placed in a 37 °C shaking water bath for 60 min. Every 10 min the tissue solution was pipetted through a 25 mL pipette (five times) and then through a 10 mL pipette (ten times). The collagenase was stopped by placing the tube on ice. The cells were passed through a 100-µm cell strainer and then centrifuged at 500× *g* for 5 min. The supernatant was removed and the cell pellet was homogenized in 10 mL dissociation buffer and centrifuged again and finally resuspended in 2 mL dissociation buffer. For the percoll gradient centrifugation a gradient was prepared by mixing 1.0 mL 10x Medium199, 12 mL dissociation buffer, and 8.6 mL percoll solution (GE Healthcare, Munich, Germany). The cell suspension was then added on top of the percoll solution and centrifuged at 30,000× *g* for 30 min at 4 °C. The tube was placed in front of a bright light source to visualize the trophoblast layer at the lower third of tube, was carefully collected with a 1 mL pipette tip, washed with 45 mL dissociation buffer, and centrifuged again. The supernatant was removed and the cells were suspended in the culture medium (Dubecco´s Modified Eagle Medium (DMEM) with 4.5 g/L D-Glucose, L-Glutamine, 25 mM HEPES, 10% FCS, 100U/mL penicillin, 100 µg/mL streptomycin, and 10 % FCS (Gibco-Invitrogen)), trypan-blue-stained and counted. Cells were snap frozen and stored at −80 °C (for proteome analysis) or seeded and cultured at 37 °C and 5% CO_2_. Cultured primary trophoblasts were stained with Haema Quick stain kit according to the manufacturer’s manual (Lehmann GmbH, Berlin, Germany). For the proteome study independent biological trophoblast preparations were used—six biological replicates and two technical replicates for *Trpv6^-/-^*; five biological replicates and three technical replicates for *wt*. Each biological sample contained the trophoblast cells isolated from 7 to 15 individual placentae at E14.5. The placentae of heterozygous *Trpv6^+/-^* embryos were not taken into account for this study.

### 4.3. RIPA Lysate Preparation and Western Blot Analysis

Mouse trophoblasts were resuspended in 500 µL RIPA buffer (150 mM NaCl, 50 mM Tris HCl, pH 8.0, 5 mM EDTA, 1% Nonidet P40, 0.1% SDS, 0.5% Na-deoxycholate, pH 7.4), homogenized with a glass Teflon potter and incubated by shaking for 45 min at 4 °C. After centrifugation at 100,000× *g* at 4 °C for 45 min the supernatant containing solubilized proteins was collected and protein concentration of the RIPA lysate was determined with the bicinchoninic acid (BCA) method (Thermo Fisher, Karlsruhe, Germany). Lysates with low protein concentrations were precipitated with 2 volumes acetone (−20 °C), centrifugated at 14,000× *g* for 10 min and washed twice with methanol. After vacuum drying the sediment was resuspended in denaturation buffer (120 mM TrisHCl, pH 6.8, 8% SDS, 20% glycerol, and 0.72 M ß-mercaptoethanol). For Western blot and Coomassie-stained gels (mass spectrometry) proteins were denatured at 60 °C for 20 min before gel electrophoresis. Western blots were performed as described [12].

### 4.4. Immunohistochemistry of MCT1 and MCT4 in the Mouse Placenta

Thawed placental cryosections (described at [1]) were washed three times in PBS for 5 min, transferred in blocking solution (0,1% TritonX100, 2% BSA (Sigma, Lot: SLBT8348), and 3% donkey serum (Jackson 017-000-121) in PBS) for 60 min at room temperature. Primary antibodies (anti-MCT1, Merck Millipore AB1286, chicken polyclonal 1:200/blocking solution, anti-MCT4, Merck-Millipore AB3546, rabbit polyclonal 1:200/blocking solution) were incubated overnight at 4 °C and secondary antibodies (goat anti-chicken 488, 1:500 and donkey anti-rabbit Cy3, 1:500) for 1 h at room temperature. Nuclear staining was done with bisbenzimide (1:10,000/PBS) for 5 min. The sections were mounted on a glass slide using fluorescence mounting medium (Flouromount-G8, Southern Biotech, Eching, Germany).

### 4.5. Antibodies

Primary antibodies directed against dysferlin (Santa Cruz, Heidelberg, Germany), GCM1 (Acris, Heidelberg, Germany), fibronectin (TF Scientific, Schwerte, Germany), granzyme (Cell Signaling, Frankfurt am Main, Germany), and β-Actin (Abcam, Cambridge, United Kingdome) were used. Fluorescence-labeled antibodies for flow cytometry analysis were obtained from ThermoFisher.

### 4.6. Growth Analysis

Four glass coverslips (diameter 10 mm) were placed in a 3.5-cm cell culture dish, overlaid with 2 mL culture medium before seeding of 200,000 primary trophoblast cells from *wt* or *Trpv6^–/–^* placentae (E14.5). Cells were incubated at 37 °C and 5% CO_2_. After 24 h, the medium was exchanged. 7 µL 0.5 M EDTA was added to some dishes to reduce the free Ca^2+^ concentration to 100 µM, calculated with MaxChelator; https://somapp.ucdmc.ucdavis.edu/pharmacology/bers/maxchelator/. Every 24 h two coverslips from each condition were fixed and stained using the Haema quick stain kit before pictures were taken randomly (Axio Imager M2, Carl Zeiss, Germany).

### 4.7. Transwell Migration Assay

A 24-well plate was equipped with transwell migration assay inserts (8.0 µm pore size, Falcon, USA). The lower chambers were filled with 1ml culture medium containing 10% FCS whereas the upper chambers contained 0.8 mL culture medium without FCS. 200,000 cells were added to the upper chamber and cultured at 37 °C and 5 % CO_2_. After 48 h, inserts were removed, cells on the upper side were carefully removed with a cotton swap, and migrated cells on the lower side of the membrane were stained with Haema quick-stain kit. The membranes were cut out and mounted with mounting medium on glass slides (DePeX, Serva, Germany). Pictures of the whole membrane were taken using a stereo microscope (M205FA, Leica, Germany). Stained areas were analyzed by using the Carl Zeiss Axio Vision LE64 program.

### 4.8. Viability Assay

Primary trophoblast cells from *wt* and *Trpv6^-/-^* placentae at E14.5 were seeded at 10,000 cells in 100 µL culture medium in one well of a 96-well plate. Cells were cultured for 7 days at 37 °C and 5% CO_2_. Every 24 h 20 µL MTS solution (Cell Titer 96 Aqueous One Solution Cell Proliferation Assay, Promega, Walldorf, Germany, USA) was added to one well and cells were incubated for 4 h at 37 °C and 5% CO_2_. The absorption of formazan was measured at λ = 490 nm using a microplate reader (Infinite M200, Tecan, Austria). 

### 4.9. Fibronectin Digestion Assay

Five micrograms of bovine fibronectin (Invitrogen, Schwerte, Germany) was incubated with 20 ng porcine trypsin (Promega, Walldorf, Germany) in 50 mM TrisHCl and 150 mM NaCl pH7.4 (TBS) for 1 h at 37 °C, inactivated by the addition of denaturing electrophoresis sample buffer for 20 min at 60 °C and then applied for Western blot analysis. Then, 100 µg protein obtained from *wt* and *Trpv6^–/–^* placentae RIPA lysates were incubated with 2.5 µg fibronectin at 37 °C (for 0.5 to 6 h) before protease activity was stopped by addition of denaturing electrophoresis sample buffer. 

### 4.10. Sample Preparation of Primary Trophoblast Lysates for Proteome Analysis

One hundred micrograms of protein obtained from trophoblast RIPA lysates was separated on NuPAGE^®^ 4–12% gradient gels (ThermoFisher, TF), fixed in the presence of 40% ethanol and 10% acetic acid and visualized with colloidal Coomassie stain (20% (*v*/*v*) methanol, 10% (*v*/*v*) phosphoric acid, 10% (*w*/*v*) ammonium sulfate, and 0.12% (*w*/*v*) Coomassie G-250) [51]. Fourteen gel pieces were cut/sampled and alternately washed twice with solution A (50 mM NH_4_HCO_3_) and solution B (50 mM NH_4_HCO_3_ and 50% (*v*/*v*) acetonitrile). Reduction of disulfide bridges was obtained by incubation at 56 °C for 30 min in 50 mM NH_4_HCO_3_ and 10 mM dithiothreitol, followed by incubation in 5 mM iodacetamide in buffer A at room temperature in the darkness for 30 min. Gel pieces were washed twice alternating with solutions A and B and dried in a vacuum centrifuge. For in-gel digestion the gel pieces were incubated in the presence of 20 μL of porcine trypsin solution (10 ng/µL, Promega) at 37 °C overnight. Resulting peptides were extracted twice by incubating the gel pieces in aqueous extraction buffer (2.5% formic acid and 50% acetonitrile) in an ultrasonic bath. Extracted peptides were collected in a fresh tube and concentrated in a vacuum centrifuge and resuspended in 20 µL of 0.1% formic acid.

### 4.11. Mass Spectrometric Measurement (Nano-LC–MS/MS)

Six microlitres of the tryptic peptides derived from each band was analyzed by online nanoflow LC–ESI–MS/MS (Ultimate 3000 RSLC nano system equipped with an Ultimate3000 RS autosampler coupled to an LTQ Orbitrap Velos Pro, (all TF, Dreieich, Germany). Peptides were trapped on a C18 trap column (75 µm × 2 cm, Acclaim PepMap100C18, 3 µm, Dionex) and separated on a reversed-phase column (nano viper Acclaim PepMap capillary column, C18; 2 µm; 75 µm × 15 cm or 75 µm × 50 cm, Dionex) at a flow rate of 200 nL/min with buffers A (water and 0.1% formic acid) and B (90% acetonitrile and 0.1% formic acid) using a gradient (4 to 55% buffer B for 56 min; 55 to 90% buffer B for 7 min). The effluent of the chromatography was directly sprayed into the mass spectrometer through a coated silica electrospray emitter (PicoTipEmitter, 30 µm, New Objective, Woburn, MA, USA) and ionized at 2.2 kV. MS spectra were acquired in a data-dependent mode. For the collision-induced dissociation (CID) MS/MS top10 method, full scan MS spectra (*m*/*z* 300–1700) were acquired in the Orbitrap analyzer using a target value of 10^6^. The 10 most intense peptide ions with charge states >2 were fragmented in the high-pressure linear ion trap by low-energy CID with normalized collision energy of 35%.

### 4.12. Raw Mass Spectrometrical Data Analysis

The fragmented tryptic peptides were identified using the MASCOT algorithm and TF Proteome Discoverer 1.4 software. Peptides were matched to tandem mass spectra by Mascot version 2.4.0 by searching of a SwissProt database (version2018_03, number of protein sequences, taxonomy mouse: 16.992). MS^2^ spectra were matched with a mass tolerance of 7 ppm for precursor masses and 0.5 Da for fragment ions. We used tryptic digest and allowed for up to two missed cleavage sites. Cysteine carbamidomethylation was set as a fixed modification and deamidation of asparagine and glutamine, acetylation of lysine, and oxidation of methionine were set as variable modifications. The MASCOT output files (.dat) were loaded in the software Scaffold (Version 4.8.8, Proteome Software Inc., Portland, OR, USA) and the proteomes were analysed with multidimensional protein-identification technology (MudPIT). To ensure significant protein identification the false discovery rate (FDR) filter was set to 5% and 1% for protein and peptide probability, respectively. The identification of two unique peptides per protein was set as the minimum for protein identification. Protein probabilities were assigned by the Protein Prophet algorithm [52]. Proteins containing similar peptides which could not be discriminated based on MS/MS analysis were grouped to satisfy the principles of parsimony. Semi-quantitative protein analysis for wild-type and *Trpv6^-/-^* trophoblast lysates were made on the basis of emPAI calculations, using no normalisation and Student’s t-test *p* < 0.05). Gene ontology (GO) term analysis was made with the Scaffold software.

### 4.13. Flow Cytometry

Cells obtained by collagenase digestion of the placenta and after subsequent percoll gradient centrifugation, respectively, were subjected to erythrocyte lysis (155 mM NH_4_Cl, 10 mM NaHCO_3_, 5 mM EDTA, pH 7.4), fixated in 1% paraformaldehyde (subsequent dilution to 0.1%) and re-suspended in PBS. PBS supplemented with 1% FCS and 1 mM EDTA was used as assay buffer, and all steps of the staining process were performed at 4 °C. For surface staining and discrimination of lymphocyte subsets, samples were stained with the following antibodies: anti-CD45-PerCP-Cy5.5 for the identification of leukocyte/lymphocyte cell populations; anti-CD3-eFluor450, anti-CD4-FITC, and anti-CD8-Pe-Cy7 for the differentiation of lymphocyte subsets (T-helper cells CD4+CD8−, cytotoxic T-cells CD4−CD8+); anti-CD45R-PE-eFluor610 for the differentiation of B lymphocytes (CD45+CD3-CD45R+); and anti-NK1.1 for the identification of NK cells (CD45+CD3-CD45R-CD4-CD8-NK1.1+). The forward scatter/ side scatter (FSC/SSC) parameters and cell gates (set using the fluorescence minus one (FMO) method) were constantly held throughout all experiments, and 100,000 cells were counted within the total cell gate (based on FSC/SSC parameters). The fluorescence signals were detected by FACS analysis (Sony SH800, Sony, Germany) and analyzed with FlowJo 10.6.2 software (Tree Star, Inc., Ashland, OR, USA).

## Supplementary Materials

Supplementary materials can be found at https://www.mdpi.com/1422-0067/21/24/9674/s1.

## Abbreviations

TRPV6	Transient receptor potential channel
ECM	Extracellular matrix
LC–MS	Liquid chromatography–mass spectrometry
FND3A	Fibronectin-domain-containing protein 3A
HTRA1	High-temperature requirement A serine protease
