# Supplementary material for: Transient Receptor Potential Vanilloid 6 (TRPV6) Proteins Control the Extracellular Matrix Structure of the Placental Labyrinth
Source: Int J Mol Sci. 2020 Dec 18;21(24):9674. doi: 10.3390/ijms21249674 (PMC7767235; doi:10.3390/ijms21249674)
Supplement: Supplementary file 1 [file ijms-21-09674-s001.zip › Supplementary Figures.docx]

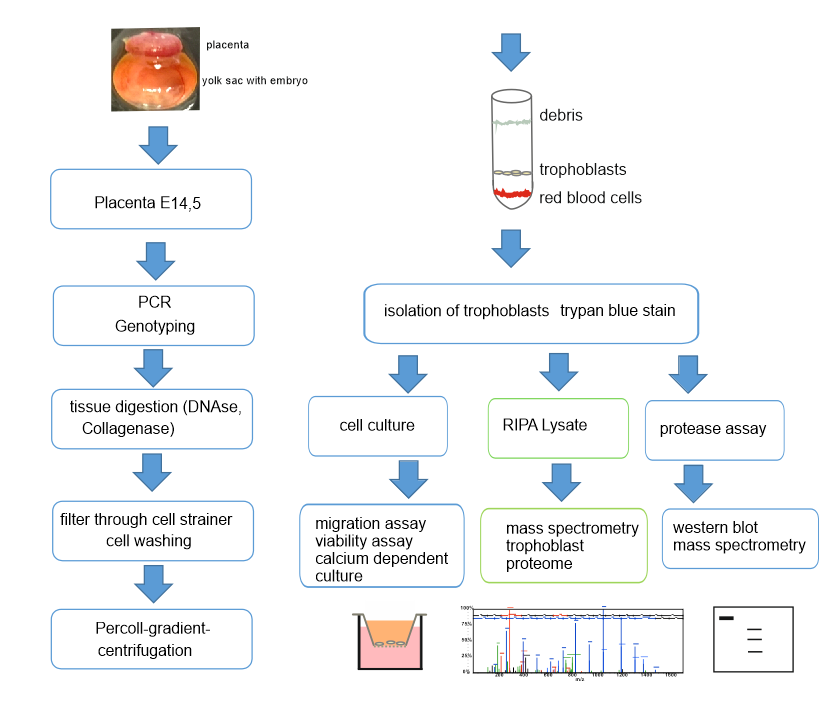


**Supplementary Figure S1.** Flow diagram: trophoblast cells were isolated from E14.5 placentae of different genotypes. After digestion, filtration and percoll gradient centrifugation, cells were isolated and brought into cell culture to investigate cell migration, viability, and calcium-dependent morphology. Alternatively, cells were collected after isolation, lysed and investigated by mass spectrometry (MS) or digestion assays were made, which were analyzed by Western blot and mass spectrometrical analysis.


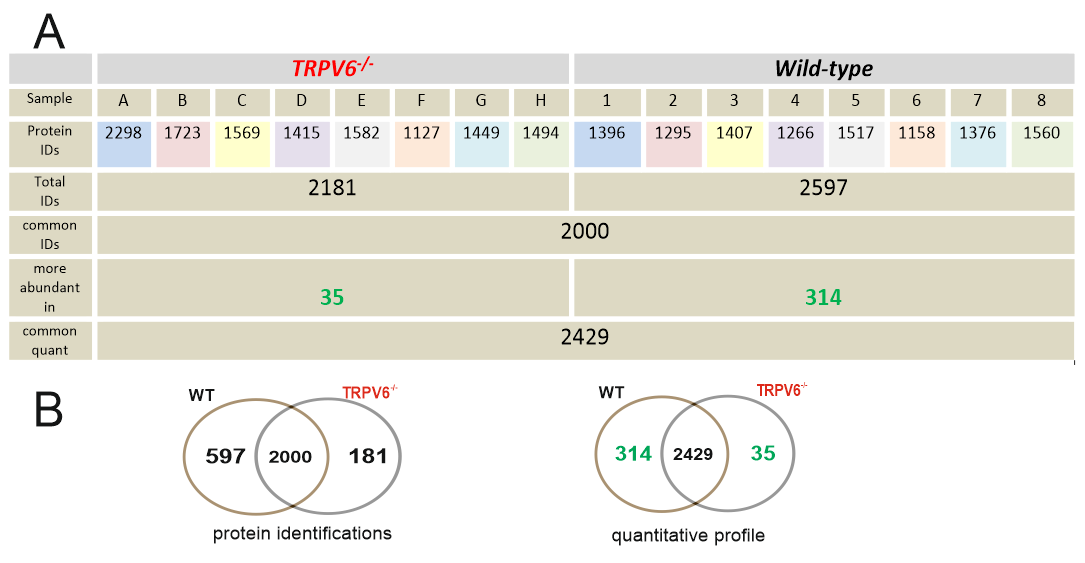


**Supplementary Figure S2.** Proteome analysis. (A) Table contains a summary of protein identifications (ID) detected in 8 *wt* and *Trpv6^-/-^* trophoblast lysates (“Protein ID”, upper row), the total protein identifications per group (n=8/genotype, =total IDs), identifications which were common in both genotypes (“Common IDs”), 314 proteins were more frequently detected in *wt* and 35 were more often detected in the KO lysates (“more abundant in”). 2429 proteins from both genotypes show no significant different quantitative profile (“common quant”). (B) Venn diagram showing qualitative and quantitative protein identifications detected in all 16 analyzed samples.


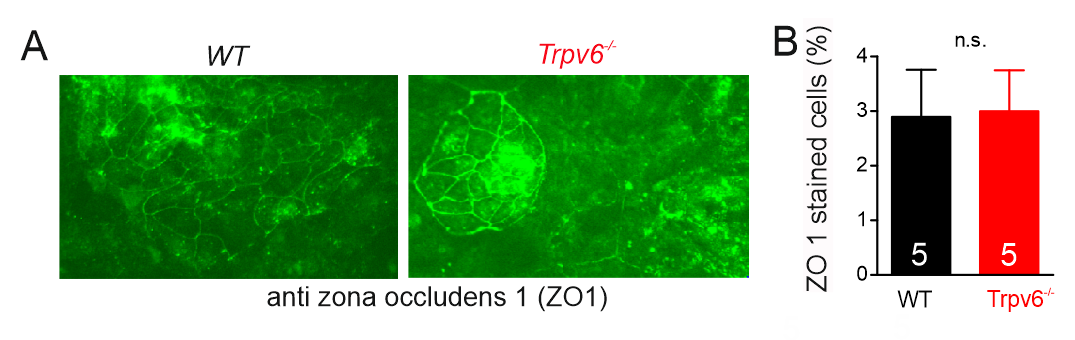


**Supplementary Figure S3.** The formation of tight junctions is not reduced in *Trpv6*-deficient trophoblasts. (**A**) Zona occludens 1 (ZO1) antibody staining of primary *wt* and *Trpv6-/-* mouse trophoblast cells at day 5 of primary cell culture. Shown here are the only two regions where a distinct ZO1 staining was visible. (**B**) The number of distinct ZO1 staining was not markedly different between the two genotypes. (n=5 coverslips (d= 1 cm)/ genotype were analyzed). The significance of the differences between both groups was calculated using Student’s t-test (*p < 0,05, ** < 0,01, *** < 0,001), Results are shown as mean + SD.


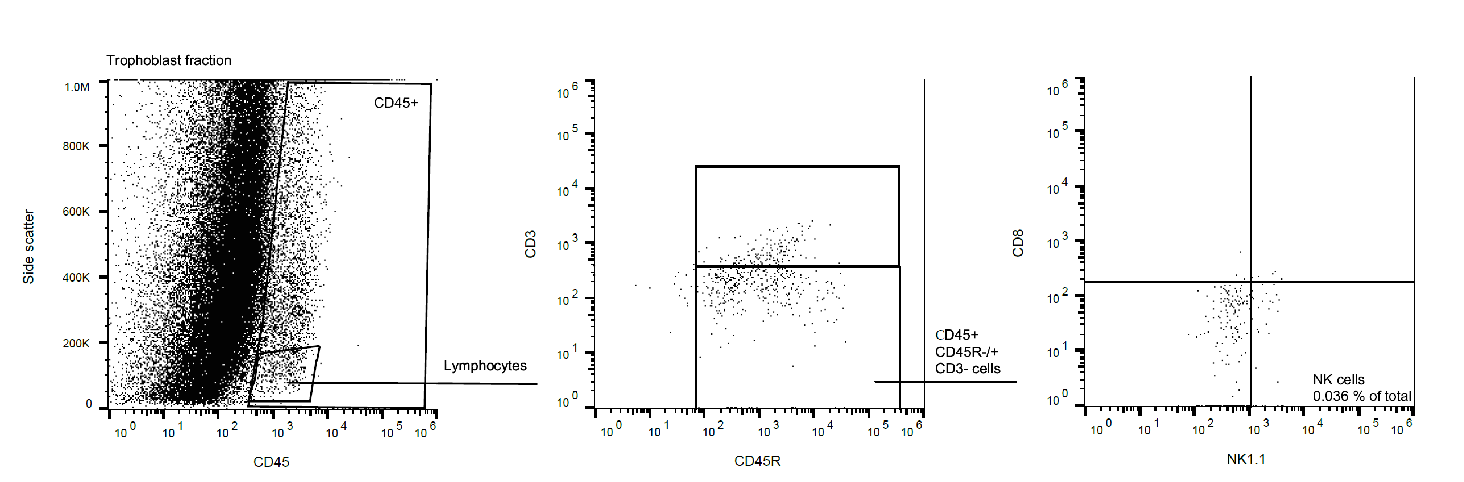


**Supplementary Figure S4.** Screening for immune cells in the trophoblast cell fraction. FACS analysis of a trophoblast cell fraction enriched from wt placentae. The percoll-gradient-enriched trophoblast fraction was subjected to antibody staining and subsequently analyzed by flow cytometry. Shown is the gating strategy as well as the frequency of detected NK cells in the total trophoblast fraction. 0.036% NK cells were detected with the NK1.1 antibody**.**
